# Serum Levels of Vasoactive Intestinal Peptide as a Prognostic Marker in Early Arthritis

**DOI:** 10.1371/journal.pone.0085248

**Published:** 2014-01-07

**Authors:** Carmen Martínez, Ana M. Ortiz, Yasmina Juarranz, Amalia Lamana, Iria V. Seoane, Javier Leceta, Rosario García-Vicuña, Rosa P. Gomariz, Isidoro González-Álvaro

**Affiliations:** 1 Departamento de Biología Celular, Facultad de Medicina, Universidad Complutense de Madrid, Madrid, Spain; 2 Servicio de Reumatología, Hospital Universitario de la Princesa, Instituto de Investigación Sanitaria la Princesa, Madrid, Spain; 3 Departamento de Biología Celular, Facultad de Biología, Universidad Complutense de Madrid, Madrid, Spain; University Hospital Jena, Germany

## Abstract

**Objective:**

Suitable biomarkers are essential for the design of therapeutic strategies in personalized medicine. Vasoactive intestinal peptide (VIP) has demonstrated immunomodulatory properties in autoimmune murine and ex vivo human models. Our aim was to study serum levels of VIP during the follow-up of an early arthritis (EA) cohort and to analyze its value as a biomarker predicting severity and therapeutic requirements.

**Methods:**

Data from 91 patients on an EA register were analyzed (76% rheumatoid arthritis (RA), 24% undifferentiated arthritis, 73% women, and median age 54 years; median disease duration at entry, 5.4 months). We collected per protocol sociodemographic, clinical, and therapeutic data. VIP levels were determined by enzyme immunoassay in sera harvested from the 91 patients (353 visits; 3.9 visit/patient) and from 100 healthy controls. VIP values below the 25^th^ percentile of those assessed in healthy population were considered low. To determine the effect of independent variables on VIP levels, we performed a longitudinal multivariate analysis nested by patient and visit. A multivariate ordered logistic regression was modeled to determine the effect of low VIP serum levels on disease activity at the end of follow-up.

**Results:**

VIP concentrations varied considerably across EA patients. Those fulfilling the criteria for RA had the lowest values in the whole sample, although no significant differences were observed compared with healthy donors. Disease activity, which was assessed using DAS28, inversely correlated with VIP levels. After a two-year follow-up, those patients with low baseline levels of VIP displayed higher disease activity and received more intensive treatment.

**Conclusion:**

Patients who are unable to up-regulate VIP seem to have a worse clinical course despite receiving more intense treatment. Therefore, measurement of VIP levels may be suitable as a prognostic biomarker.

## Introduction

Rheumatoid arthritis (RA) is a systemic autoimmune disease with a heterogeneous clinical spectrum. At present, our ability to predict this heterogeneity is poor, likely because of limitations in understanding its molecular complexity. Recent efforts to improve outcome in patients with RA have focused on the early stages of the disease [1], when aggressive treatment can slow progression and change long-term course. Currently, another crucial therapeutic goal is to delay/prevent progression of undifferentiated arthritis (UA) and one of the challenges faced by rheumatologists is the classification of these patients. Thus, many studies have attempted to identify prognostic markers in early RA or UA that correlate with disease progression in order to establish which patients are at risk for poor outcome or, conversely, which are destined to have a more benign disease so that overtreatment can be avoided [2]. However, criteria for this clustering of patients are scarce. To date, validated biomarkers of severity such as rheumatoid factor (RF) or anti-citrullinated peptide antibodies (ACPA) do not enable us to identify those patients who require more intensive treatment. Although challenging, identification of such predictive tools would help us tailor treatment.

Vasoactive intestinal peptide (VIP) is produced by neural, endocrine, and immune cells. It has anti-inflammatory and immunoregulatory effects [3], which are mediated by three G-protein–coupled receptors (VPAC1, VPAC2, and PAC1) [4], [5]. First studies evaluating the endogenous role of VIP were performed in transgenic mice harboring a chimeric VIP gene which showed reduced brain VIP content and deficiencies in learning abilities supporting an important function for VIP in vivo [6]. Recently, it has been described that VIP-deficient mice display certain physiological abnormalities [7], [8] and exhibit reduced mortality and impaired proinflammatory responses to lipopolysaccharide-induced endotoxemia [9], suggesting that defects in the innate arm of immunity may occurs in the chronic absence of VIP. Numerous reports have focused on the effects of VIP treatment. Thus, administration of VIP has demonstrated therapeutic effects in several murine models of inflammatory/autoimmune diseases [10]–[14]. In a murine model of collagen-induced arthritis, administration of VIP reduced joint inflammation and destruction, thus decreasing the inflammatory response and inducing a shift in the Th1/Th2 balance [10], [15]. Research in human models has confirmed the modulatory effects of VIP in *ex vivo* assays with fibroblast-like synoviocytes and peripheral blood lymphocytes from patients with RA through down-regulation of proinflammatory mediators [16]–[18]. Decreased expression of VIP was recently reported in the synovial fluid of patients with osteoarthritis (OA) and poor radiological progression, indicating a protective role for VIP [19]. Moreover, patients with juvenile idiopathic arthritis have lower serum levels of VIP than healthy controls [20].

Consequently, the role of endogenous VIP in the pathophysiology of RA is becoming clearer. Our hypothesis is that VIP serum levels are correlated with disease severity. Therefore, the objective of this work was to assess VIP levels during the follow-up of patients with early arthritis (EA) and to explore its potential value as a biomarker in RA.

## Methods

### Ethics Statement

The register protocol was reviewed and approved by the Ethics Committee for Clinical Research at the Instituto de Investigación Sanitaria La Princesa. All patients were informed about the study and signed an informed consent form before inclusion in the EA register.

### Patients and Controls

The study sample comprised 91 patients enrolled on our EA register. The inclusion criteria included more than 1 swollen joint for at least 4 weeks and symptoms for less than a year. Only data from patients fulfilling the 1987 American College of Rheumatology criteria for RA [21] within the 5-year follow-up (n = 69) or with chronic undifferentiated arthritis (n = 22) were analyzed. Patients with diagnosis of spondyloarthritis, connective tissue diseases or crystal induced arthritis, were excluded from the analysis. The register protocol included 5 visits (baseline, 6, 12, 24, and 60 months), and at each one we recorded the following data in an electronic database: clinical and demographic information; disease duration at the beginning of follow-up; 28-joint Disease Activity Score (DAS28) [22]; global disease activity on a 100-mm visual analogue scale assessed by both the patient and the physician; Health Assessment Questionnaire score (HAQ; Spanish version) [23]; and the results of laboratory tests including erythrocyte sedimentation rate (ESR), C-reactive protein (CRP), rheumatoid factor (RF, assessed by nephelometry; positive >20 IU/ml), and anti-citrullinated peptide antibody (ACPA, measured by enzyme immunoassay [EIA]: Euro-Diagnostica Immunoscan RA; positive >50 IU/ml).

Healthy donors (n = 100) were recruited from the Centro de Transfusiones de la Comunidad de Madrid. Following the Spanish Personal Data Protection Law, their demographic information was confidential.

### Measurement of Serum VIP

VIP levels were determined using a competitive EIA with a commercially available kit according to the manufacturer's instructions (Phoenix Pharmaceutical, Karlsruhe, Germany). We previously optimized sample preparation procedure to obtain optimal results. First, it was tested the extraction of peptides from serum using a SEP-COLUMN containing 200 mg of C18, according to the manufacturer’s recommendations. Second, serum samples were concentrated by using a lyophilizer to assay different concentrations. Then, VIP levels were determined. The best results were obtained in reconstituted dried extract (2∶1) without the use of columns. The assay was performed as follow, briefly, the samples were freeze-dried and dissolved in EIA buffer (2∶1), added to an immunoplate pre-coated with a secondary antibody, and incubated with biotinylated VIP and a primary antibody whose Fab fragment competitively binds to the biotinylated peptide and targeted peptide in samples. After washes, the wells were incubated with streptavidin-horseradish peroxidase, which catalyzed the oxidation of the substrate solution. The reaction was stopped using the stop solution and absorbance measured at 450 nm. A standard curve of known concentration was established. The concentration in the samples was determined by extrapolation to this curve and by applying the corresponding dilution factor. Samples from each patient were assayed twice. The minimum detectable concentration was 0.12 ng/ml of sample, with an intra-assay and interassay variation of ≤5% and 15%, respectively.

Since serum samples were stored at –80°C for different periods of time until assay (range, 1 to 112 months), we first analyzed the effect of sample frozen-time on the ability of our assay to detect VIP. As shown in Figure S1, no significant correlation was observed between VIP serum level and frozen-time.

### Statistical Analysis and New Variables

Normally distributed quantitative variables were expressed as the mean ± standard deviation, while non-normally distributed variables were expressed as the median and interquartile range (IQR). Qualitative variables were described using an estimation of the proportions. Variables with a normal distribution were analyzed using the *t* test, while the Mann-Whitney or Kruskal-Wallis tests were used for variables with a non-normal distribution. A χ^2^ test or the Fisher exact test was used to compare categorical variables.

Considering that the raw data contained considerably left-shifted values (Figure S2A), we decided to censor values higher than 1000 pg/ml in this figure (Figure S2B) to avoid missing information if patients with very high VIP values were excluded. In addition, since the censored variable does not show a Gaussian distribution, data were normalized through logarithmic transformation (Figure S2C).

Additional variables were defined to further describe the role of VIP in the progress of arthritis. VIP levels were considered to be low when the concentration was below the 25^th^ percentile of its concentration in the healthy population (370 pg/ml). The intensity of disease-modifying anti-rheumatic drugs (DMARDs) treatment (IDT) was assessed as the number of days of treatment with each DMARD during follow-up adjusted for weighted coefficients, as described elsewhere [24].

To identify factors that influenced VIP levels during follow-up (logarithmic transformation of the censored VIP levels as the dependent variable), we used data from 340 visits in the 88 patients with all information available in at least 2 visits to fit a population-averaged model by means of generalized linear models nested by patient and visit using the *xtgee* command of Stata 12 for Windows (StataCorp LP, College Station, Texas, USA). The population-averaged generalized estimating equations were first modeled by adding all variables with a *p* value <0.15 in the bivariate analysis. The final models were constructed using a quasi-likelihood estimation based on the independence model information criterion [25] and Wald tests, after removing all variables with *p*>0.15.

To determine the usefulness of baseline VIP levels for predicting disease progress, we performed an ordered logistic model using the *ologit* command of Stata 12. The dependent variable was the disease activity level at the 2-year follow-up, using the cut-off points for DAS28 proposed by Prevoo et al. (<2.6, remission; 2.6 to 3.2, low disease activity; 3.2 to 5.1, moderate disease activity; >5.1 high disease activity) [22]. In this model, the dependent variable considered remission as 0 and low, moderate, and high disease activity as 1, 2, and 3. Thus, the ordered logistic analysis can also estimate cut-off points that aid the interpretation of the coefficients for each independent variable according to the levels of the dependent variable. The analysis was modeled as described above for *xtgee.*

The significance level was set at p<0.05.

## Results

### Characteristics of Patients with EA. VIP Levels in Patients and Healthy Donors

A total of 91 patients with RA (n = 69) or UA (n = 22) were included in the study. The main difference between these two subgroups was a higher frequency of female patients in the RA group, in which more severe disease at baseline and a higher prevalence of RF and ACPA positivity were also found (Table 1). No significant differences were detected between the groups in variables expressing a subjective component such as pain, global disease assessment by patient, tender joint count, and HAQ.

**Table 1:** Baseline characteristics of patients with early arthritis.

	Rheumatoid artritis(n = 69)	Undifferentiated arthritis(n = 22)	Total(n = 91)	P value
Age (years)	54 [45–66]	55 [44–69]	54 [45–66]	n.s.
Female gender (%)	78	54	73	0.004
Disease duration (months)	5 [3]–[8]	7 [3]–[11]	5.4 [3.2–8.4]	n.s.
GDAPa	46 [29–60]	46 [28–58]	46 [28–60]	n.s.
GDAPh	50 [30 - 72]	30 [14–50]	42 [25–66]	0.002
VAS Pain	52 [25–70]	48 [13–60]	50 [20–70]	n.s.
HAQ	1.125 [0.625–1.750]	0.875 [0.375–1.375]	1 [0.625–1.625]	n.s.
TJC	6 [2]–[12]	3 [0–10]	6 [1]–[12]	n.s.
SJC	7 [3]–[10]	3 [0–6]	5 [2]–[9]	0.004
ESR	32 [19–54]	17 [8–34]	30 [15–46]	0.006
CRP (mg/dl)	0.92 [0.2–1.96]	0.48 [0.1–0.82]	0.72 [0.16–1.7]	0.047
DAS28 (0–10)	5.0 [4.0–6.1]	3.6 [3.1–4.5]	4.8 [3.5–6.0]	0.002
RF (%)	54	18	45	0.004
ACPA+ (%)	60	14	49	<0.001
VIP (pg/ml)	342 [262–440]	375 [243–490]	352 [261–444]	n.s.

Data are shown as the median or percentage. GDAPa: global disease assessment by patient; GDAPh: global disease assessment by physician; VAS: visual analog scale; HAQ: health assessment questionnaire; TJC: tender joint count; SJC: swollen joint count; ESR: erythrocyte sedimentation rate; CRP: C-reactive protein; DAS28∶28-joint count Disease Activity Score; RF: rheumatoid factor; ACPA: anti-citrullinated peptide antibodies; VIP: vasoactive intestinal peptide. n.s.: not significant.

**Figure 1:**
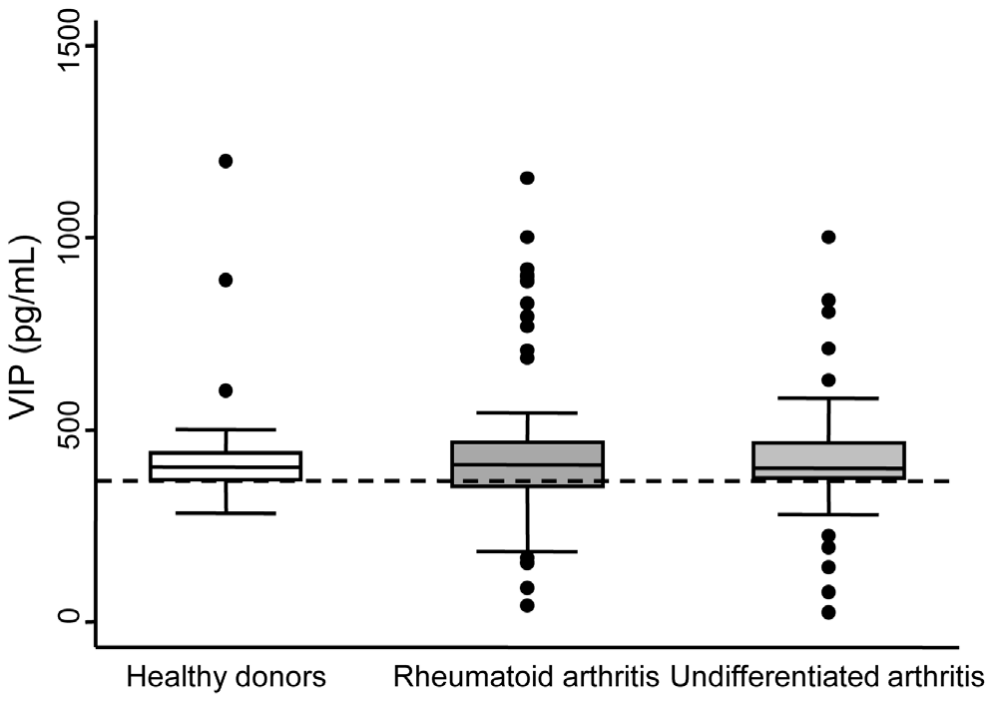
Variability of VIP levels in healthy controls (n = 100) and patients with early arthritis (Rheumatoid arthritis n = 69, Undifferentiated arthritis n = 22). Data are presented as the interquartile range (p75 upper edge of the box, p25 lower edge, p50 midline), p90 (line above the box), and p10 (line below the box) of the serum VIP levels. Dots represent outliers. Dashed lines show the 25^th^ percentile of the healthy donor group.

Considering serum levels of VIP at baseline, no significant differences were detected between the groups (Table 1) or between patients and healthy controls (Figure 1). Nevertheless, the distribution of the VIP serum concentration was considerably heterogeneous, especially among RA patients, and ranged from 100 to >1000 pg/ml. In addition, the lowest levels of VIP tended to be more frequent in RA and UA patients than in healthy donors (Figure 1).

### Low VIP Levels are Associated with Higher Disease Activity in Patients with EA

**Figure 2:**
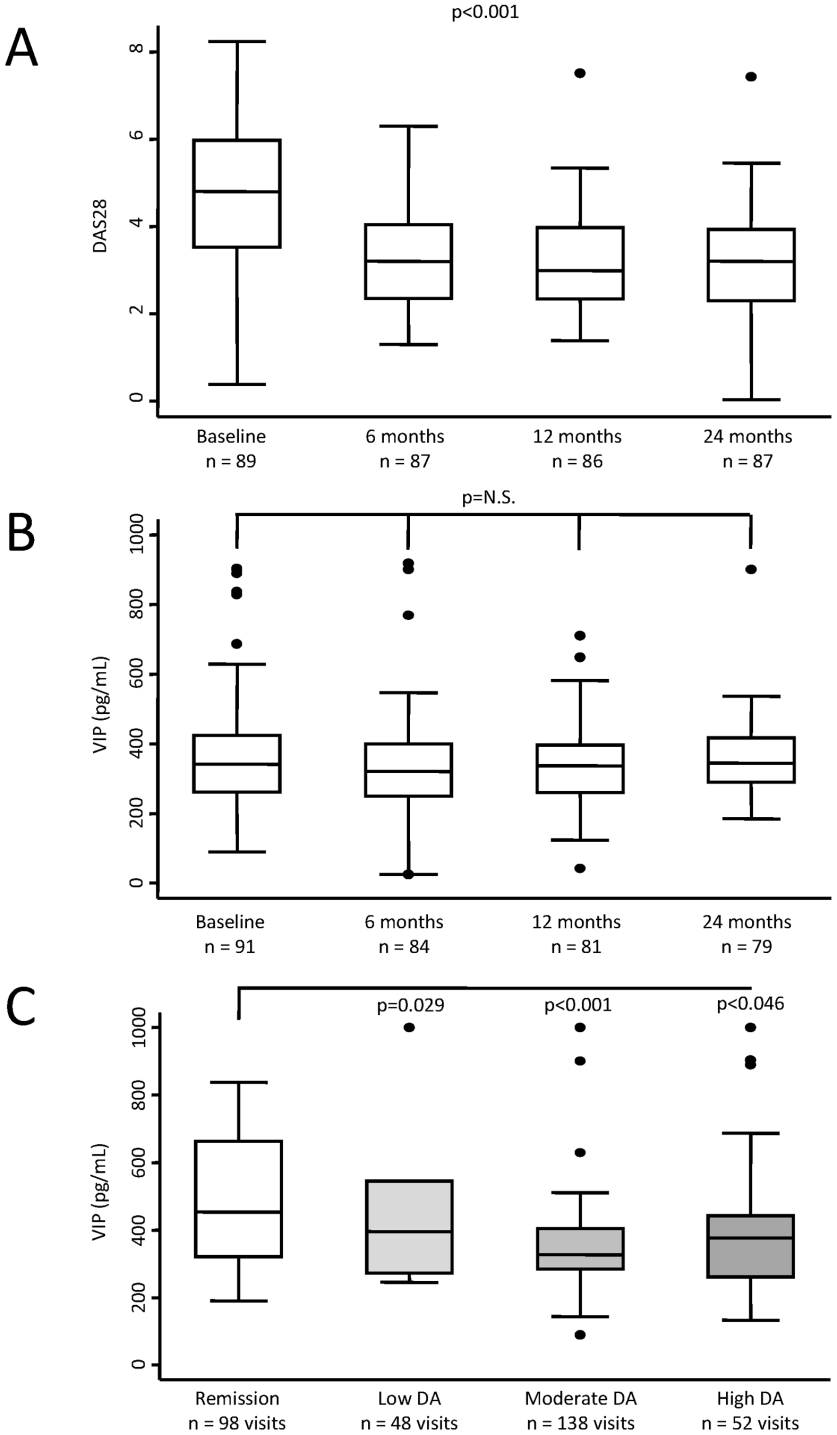
VIP serum levels correlate inversely with disease activity in patients with early arthritis. **A)** Progress of disease activity estimated by DAS28 at follow-up visits. **B)** Serum levels of VIP during the follow-up period. **C)** Serum VIP levels considering disease activity level. Data are presented as the interquartile range (p75 upper edge of the box, p25 lower edge, p50 midline), p90 (line above the box), and p10 (line below the box) of the serum VIP levels. Dots represent outliers. Statistical significance was established using the Kruskal-Wallis test in panels A and B. In panel C the significance level is that obtained in the multivariable analysis displayed in Table 2, model 2.

Despite a significant decrease in disease activity during follow-up (Figure 2A), we could not detect a parallel down-regulation of serum VIP levels (Figure 2B). Therefore, we performed a multivariate analysis to achieve a more accurate appraisal of the potential relationship between VIP levels and disease activity in patients with EA. Our data showed that elderly patients expressed slightly but significantly higher serum levels of VIP during follow-up (Table 2 and Figure S3). In addition, VIP levels tended to be significantly higher in visits at which patients were treated with TNF blockers. Interestingly, after adjustment for these confounders, we observed a significant and negative correlation between disease activity, estimated through DAS28, and VIP serum levels (Table 2, model 1).

**Table 2:** Variables associated with VIP serum levels during follow-up of patients with early arthritis.

	Model 1		Model 2	
	β coeff ± SE	P	β coeff ± SE	P
Gender				
Male	Reference		Reference	
Female	0.108±0.067	0.106	0.105	0.086
Diagnosis				
RA	n.i.	n.s.	n.i.	n.s.
UA				
ACPA	n.i.	n.s.	n.i.	n.s.
Age (by 10 yr)	0.05±0.019	0.013	0.05±0.018	0.008
GDA Pat	n.i.	n.s.	n.i.	n.s.
GDA Phy	n.i.	n.s.	n.i.	n.s.
HAQ	n.i.	n.s.	n.i.	n.s.
SJC	n.i.	n.s.	n.i.	n.s.
MS	n.i.	n.s.	n.i.	n.s.
DAS28	−0.027±0.014	0.045	–	–
Disease activity				
Remission	–	–	Reference	
Low			−0.094±0.043	0.029
Moderate			−0.187±0.049	<0.001
High			−0.113±0.057	0.046
Leflunomide(mg/d)	n.i.	n.s.	n.i.	n.s.
TNF blockers	0.183±0.075	0.01	0.165±0.065	0.011

The longitudinal analysis was performed with data (logarithmic transformation of censored VIP levels; see figure S2) from 340 visits corresponding to the 88 patients with all information available in, at least, two visits. Model 1 was fitted using the continuous value of DAS28 as measure of disease activity while model 2 includes the categorical variable based in cut-off values of this index. The average number of visits by patient was 3.9. The table shows all the variables reaching p<0.15 at the bivariate analysis (see Methods section for further information on multivariable analysis modeling). Coeff: coefficient; CI: confidence interval; RA: rheumatoid arthritis; UA: undifferentiated arthritis; ACPA: anti-citrullinated peptide antibodies; yr, year; GDA: global disease assessment; Pat: patient; Phy: physician; HAQ: health assessment questionnaire; SJC: swollen joint count; MS: morning stiffness; DAS28, 28-joint Disease Activity Score; TNF: tumor necrosis factor; n.s.: not significant; n.i.: not included.

Next, we analyzed the concentration of VIP at the different visits after clustering the results in four groups: remission, low, moderate, and high disease activity. As shown in Figure 2C, we observed lower levels of VIP in those visits with moderate or high activity compared to visits at which patients were in remission and those differences reached statistically significance after adjusting by confounders (Table 2, model 2). However, this approach did not reveal whether inflammation induced low VIP levels or those patients with low VIP showed more intense disease activity.

### Low Baseline VIP Serum Levels as a Biomarker of Disease Severity

**Figure 3:**
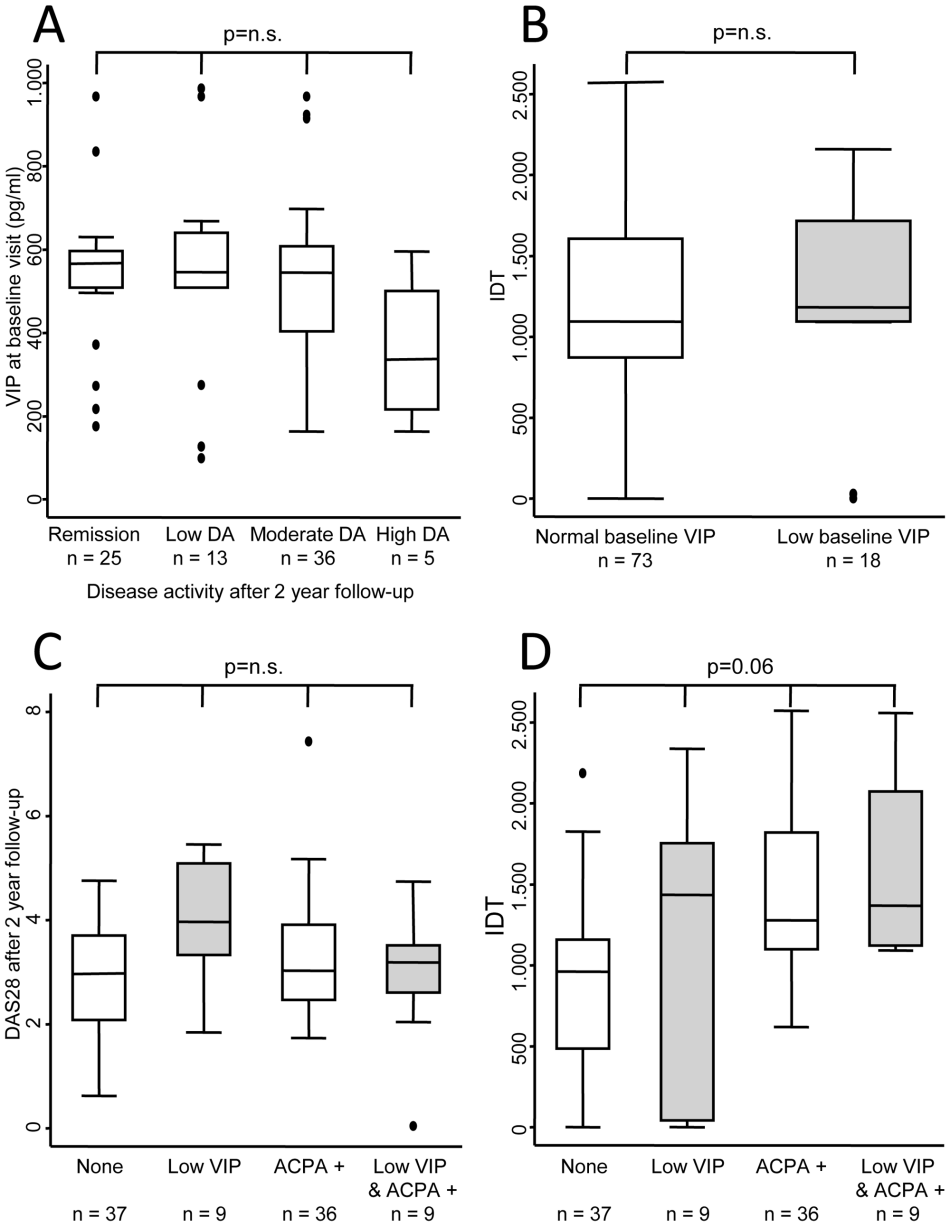
Serum VIP level as a prognostic marker. **A)** VIP levels at baseline according to the degree of disease activity after two years of follow-up. **B)** Intensity of treatment in two subpopulations of early arthritis patients clustered according to serum VIP level. Cumulative DMARD treatment during the follow-up period was estimated using the intensity of DMARD treatment (IDT) variable (see Methods)**. C)** Disease activity estimated by DAS28 after two years of follow-up in a population of patients with early arthritis according to VIP levels and the presence of anti-citrullinated peptide antibody (ACPA+). **D)** Intensity of DMARD treatment in different subpopulations clustered by serum VIP levels and the presence of ACPA. Data are presented as the interquartile range (p75 upper edge of the box, p25 lower edge, p50 midline), p90 (line above the box), and p10 (line below the box). Dots represent outliers. Statistical significance was established using the Kruskal-Wallis test in panels A, C and D or Mann-Whitney test in panel B.

Next, we studied whether having low VIP serum levels at baseline could predict long-term disease activity. As Figure 3A shows, those patients who continued to have high or moderate disease activity levels after a two-year follow-up had lower baseline VIP serum levels. This behaviour was detected despite the observed trend toward higher prescription of DMARDs in patients with low VIP serum levels (Figure 3B).

In view of the various confounding factors that could influence disease activity level after two years of follow-up, we performed a multivariate ordered logistic regression to determine which variables contribute to an increased level of disease activity. Female patients showed an odds ratio (OR) of 4.3 for poorer disease outcome than male patients (Table 3). In addition, patients requiring combined therapy displayed an OR of 6.6 for remaining at a higher disease activity level than those who did not require DMARDs. After adjustment for these confounders, having low baseline VIP levels was associated with a higher level of disease activity after two years of follow-up. However, marked interference with ACPA positivity was observed (Table 3). Thus, patients with low VIP serum levels but negative ACPA had an OR of 6.1 for being at a higher disease activity level than those with negative ACPA levels and normal VIP serum levels (Table 3 and Figure 3C). By contrast, patients with positive ACPA levels showed a nonsignificant trend toward lower disease activity regardless of VIP levels (Table 3 and Figure 3C). In addition, the model revealed that the effect of gender, treatment, and baseline VIP level was more intense when the level of disease activity increased (β coefficients for cutpoints in Table 3).

**Table 3:** Effect of low baseline VIP levels in serum on disease activity after a two-year follow-up.

	Odds ratio (95% CI)	P
Age (years)		
<45	Ref.	
45–65	–	n.s.
>65	–	n.s.
Gender		
Man	Ref.	
Woman	4.35 (1.46–12.99)	0.008
Diagnosis		
RA	Ref.	
UA	–	n.s.
Positive RF	–	n.s.
Interaction VIP, ACPA		
Normal VIP, ACPA-negative	Ref.	
Low VIP, ACPA-negative	6.11 (1.28–29.22)	0.023
Normal VIP, ACPA-positive	0.68 (0.24–1.9)	0.458
Low VIP, ACPA-positive	0.24 (0.03–1.85)	0.170
DMARD treatment		
None	Ref.	
Monotherapy	2.2 (0.30–12.96)	0.437
Combined therapy	6.62 (0.84–51.93)	0.071
Baseline DAS28	–	n.s.
Cutpoints	β coeff. (95% CI)	
Remission/Low disease activity	1.59 (–0.40 to 3.59)	–
Low/Moderate disease activity	2.46 (0.44–4.49)	–
Moderate/High disease activity	5.97 (3.57–8.38)	–

DAS28∶28-joint Disease Activity Score; HAQ: Health Assessment Questionnaire; Coeff.: coefficient; Ref.: reference; RA: rheumatoid arthritis; UA: undifferentiated arthritis; ACPA: anti-citrullinated peptide antibody; n.s.: not significant.

In terms of treatment, ACPA positivity was associated with higher prescription of DMARDs, as occurred when low VIP levels were associated with ACPA positivity (Figure 3D). More heterogeneous regimens were found in patients with low VIP levels (treating physician blinded) and negativity for ACPA, but they undoubtedly received more intensive treatment than the population that met neither of these conditions (Figure 3B and 3D).

## Discussion

Rheumatoid arthritis is a complex heterogeneous disease resulting from the interaction between genetic and environmental triggers and from the intervention of key molecules that modulate its severity. To date, there are no published data demonstrating an association between abnormal regulation of VIP and an increased risk for developing RA. However, growing evidence supports the ability of VIP to regulate the intensity of the inflammatory process and the immune response that contribute to the pathogenesis of rheumatic diseases [26]. In this work, we showed that the lowest values of serum VIP levels were clustered in the EA group, despite no significant differences compared with healthy donors. Furthermore, our results reveal an inverse correlation between disease activity and VIP concentration in serum. Thus, patients with lower VIP levels showed higher DAS28 scores, and, conversely, higher VIP levels were detected in the group of patients in remission and in those with significantly weaker disease activity.

Several experimental findings support this observation. Treatment with VIP reduced the incidence and severity of arthritis in murine models of RA by decreasing the production of proinflammatory cytokines and chemokines, and inducing a shift in the Th phenotype from a Th1-type toward a Th2-type response and generating efficient regulatory T cells [10], [27]. In human fibroblast-like synoviocytes from RA patients, VIP downregulated the expression and production of proinflammatory cytokines, chemokines and COX2 as well as the production of IFNβ, CXCL8, and the matrix metalloproteinase-3 induced by TLR ligands [16], [17], [28].

Given the above evidence on the role of VIP, it seems reasonable that patients in whom production of this immunoregulatory peptide did not increase have more severe autoimmune and inflammatory responses and poor outcomes. In close correlation with our data, expression of VIP in synovial fluid and articular cartilage from patients with OA was negatively associated with progressive joint damage as a potential indicator of disease severity, thus suggesting that VIP could play a protective role in progression of OA [19]. Moreover, low serum levels of VIP have been described in patients with juvenile idiopathic arthritis who show clinical evidence of cardiac autonomic neuropathy associated with a parasympathetic dysfunction. In these patients, a significant positive association was found between cardiac autonomic neuropathy and major disease manifestations, including activity; therefore, the authors suggested that serum VIP should be assessed in these patients [20].

Although several parameters (eg, RF, ACPA, ESR, and CRP) have been proposed as predictors of long-term outcome of EA, they enable us to classify only 65% of patients [2], [24], [29]–[31]. Furthermore, as RF and ACPA largely overlap, identifying new prognostic markers would be essential for providing complementary information in order to improve the performance of a biomarker-guided strategy. In this setting, the association between VIP levels and disease activity reported here suggests that baseline serum VIP could be a feasible biomarker that enables EA patients to be stratified for therapeutic decision making. In fact, our data support that measurement of serum VIP levels at baseline enhances the predictive value of ACPA in determining long-term outcome in patients with EA. Thus, ACPA-negative patients with low VIP serum levels had a higher degree of disease activity than ACPA-negative patients with normal serum VIP levels and an even higher degree of disease activity than ACPA-positive patients. This finding likely reflects selection bias, since the presence of ACPA, a recognized marker of poor prognosis to which the attending rheumatologist was not blinded, was associated with more intense prescription of DMARDs. As a consequence, ACPA-positive patients with normal VIP showed similar disease activity to ACPA-negative patients with normal VIP. Interestingly, although the difference was not significant, ACPA-positive patients with low VIP serum levels had a slightly higher degree of disease activity and a greater requirement for DMARDs than ACPA-positive patients with normal VIP serum levels. On the other hand, ACPA-negative patients with low VIP levels received more heterogeneous treatment, probably because of the lack of a suitable marker, since physicians were entirely unaware of the value of this parameter.

An additional advantage of VIP as a prognostic marker is its scarce variation during follow-up, which indicates that VIP levels depend on the individual and do not change owing to external factors during follow-up. Thus, VIP concentrations could predict the outcome of arthritis independently of the treatment prescribed and the phase of the disease.

Our study is subject to a series of limitations. First, our data show that elderly patients have slightly but significantly higher serum levels of VIP. However, as demographic information in the healthy volunteers was confidential, we were unable to draw a comparison with the patients. In this regard, the correlation between VIP levels and gender and age has received little attention in the literature. The only study that does examine this correlation found no differences in VIP concentrations between men and women with secondary hyperparathyroidism undergoing dialysis [32]. Nevertheless, variation attributable to age in our study is lower than individual variability among EA patients (Figure S3). Second, the absence of a pre-established treatment strategy in our unit means that variations in the prescription of DMARDs by the rheumatologists involved in the study could interfere with the results. Third, considering the dispersion of VIP values owing to the variability of ELISA, we had to apply mathematical modifications to optimize the statistical analysis, thus hampering implementation in daily clinical practice.

Our study has the following strengths. First, exhaustive and rigorous collection of data on treatment with DMARDs goes some way to resolving the second limitation. VIP levels were measured retrospectively; therefore, the rheumatologists were blinded to serum VIP levels but not to ACPA reactivity when they selected therapy. Consequently, they were able to prescribe more intensive treatment to ACPA-positive patients. Second, we included a high number of samples and clinical data (about 4 visits per each of the 91 patients).

In conclusion, our study demonstrates that EA patients with low baseline VIP levels have worse disease outcome. Validation of the predictive value of serum VIP levels would enable earlier and more intensive treatment of patients with the most aggressive forms of RA.

## Supporting Information

Figure S1 **Frozen storage does not affect the measurement of serum VIP levels.** VIP concentration at serum from samples stored from one month to 10 years is shown. The red line represents the linear prediction obtained with the command *lfit* of Stata 12.
(TIF)

Figure S2 **Normalization of the VIP serum levels variable in order to obtain a distribution closer to Gaussian.**
(TIF)

Figure S3 **Correlation between age and VIP serum levels.** Data are shown as dot plot and the exponential linear prediction with 95% confidence interval at ages 20, 30, 40, 50, 60, 70 and 80. These data were obtained using the command *marginsplot* of Stata 12 after performing the multivariable analysis displayed in Table 2 (dependent variable logarithmic transformation of censored VIP).
(TIF)
