# Supplementary figures and images for: Serum Levels of Vasoactive Intestinal Peptide as a Prognostic Marker in Early Arthritis
Source: PLoS One. 2014 Jan 7;9(1):e85248. doi: 10.1371/journal.pone.0085248 (PMC3883710; doi:10.1371/journal.pone.0085248)

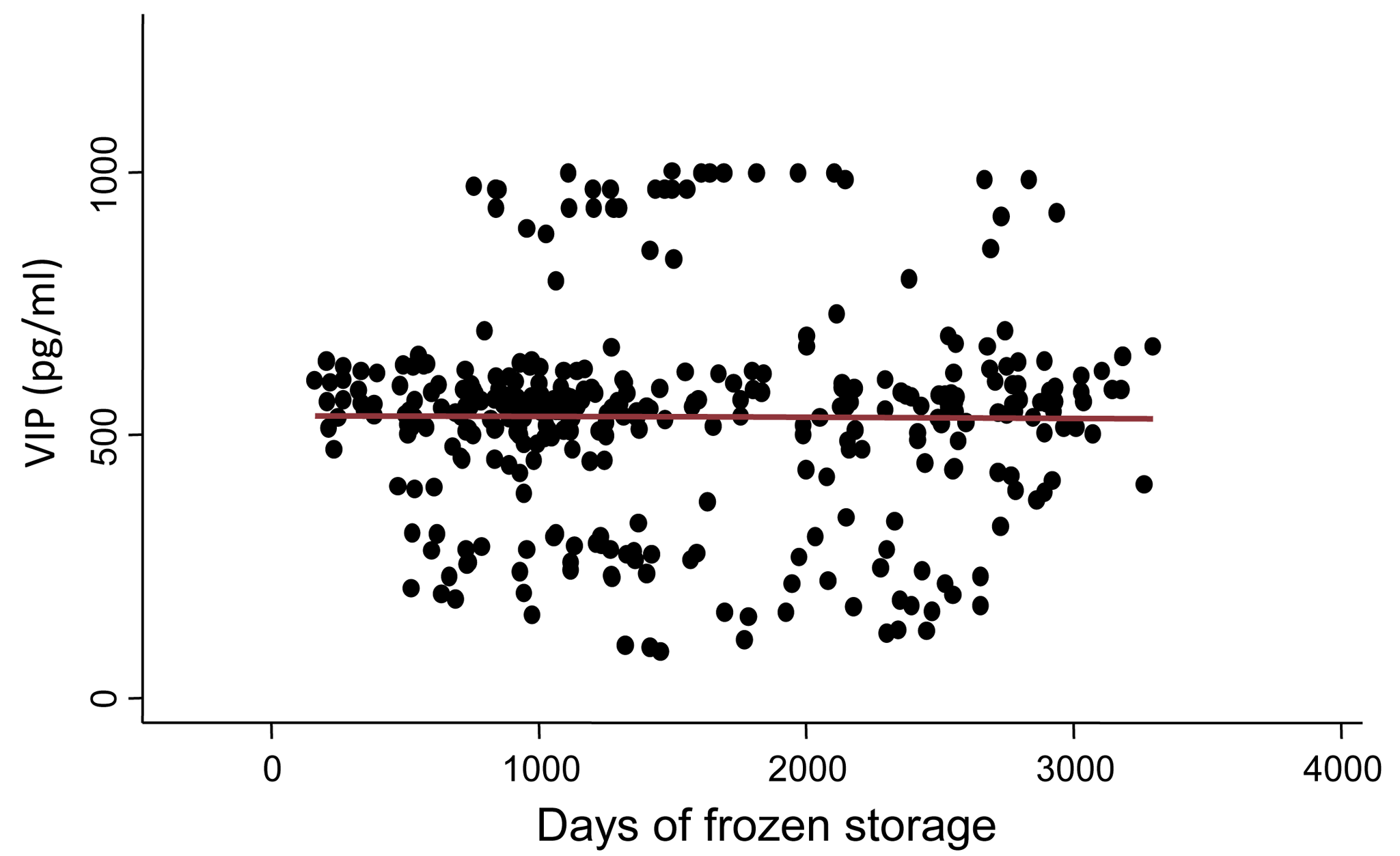

Supplement: Figure S1 — Frozen storage does not affect the measurement of serum VIP levels. VIP concentration at serum from samples stored from one month to 10 years is shown. The red line represents the linear prediction obtained with the command lfit of Stata 12. (TIF) [file pone.0085248.s001.tif]

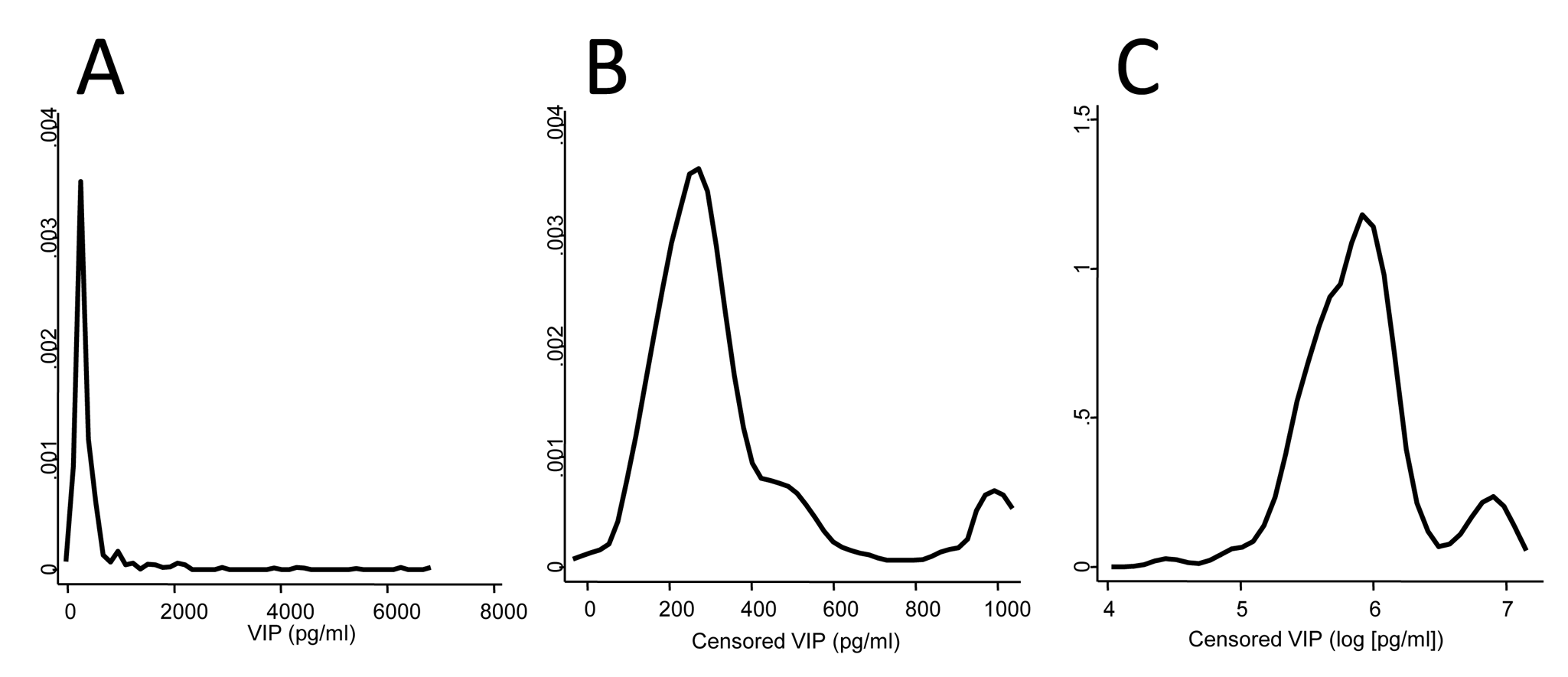

Supplement: Figure S2 — Normalization of the VIP serum levels variable in order to obtain a distribution closer to Gaussian. (TIF) [file pone.0085248.s002.tif]

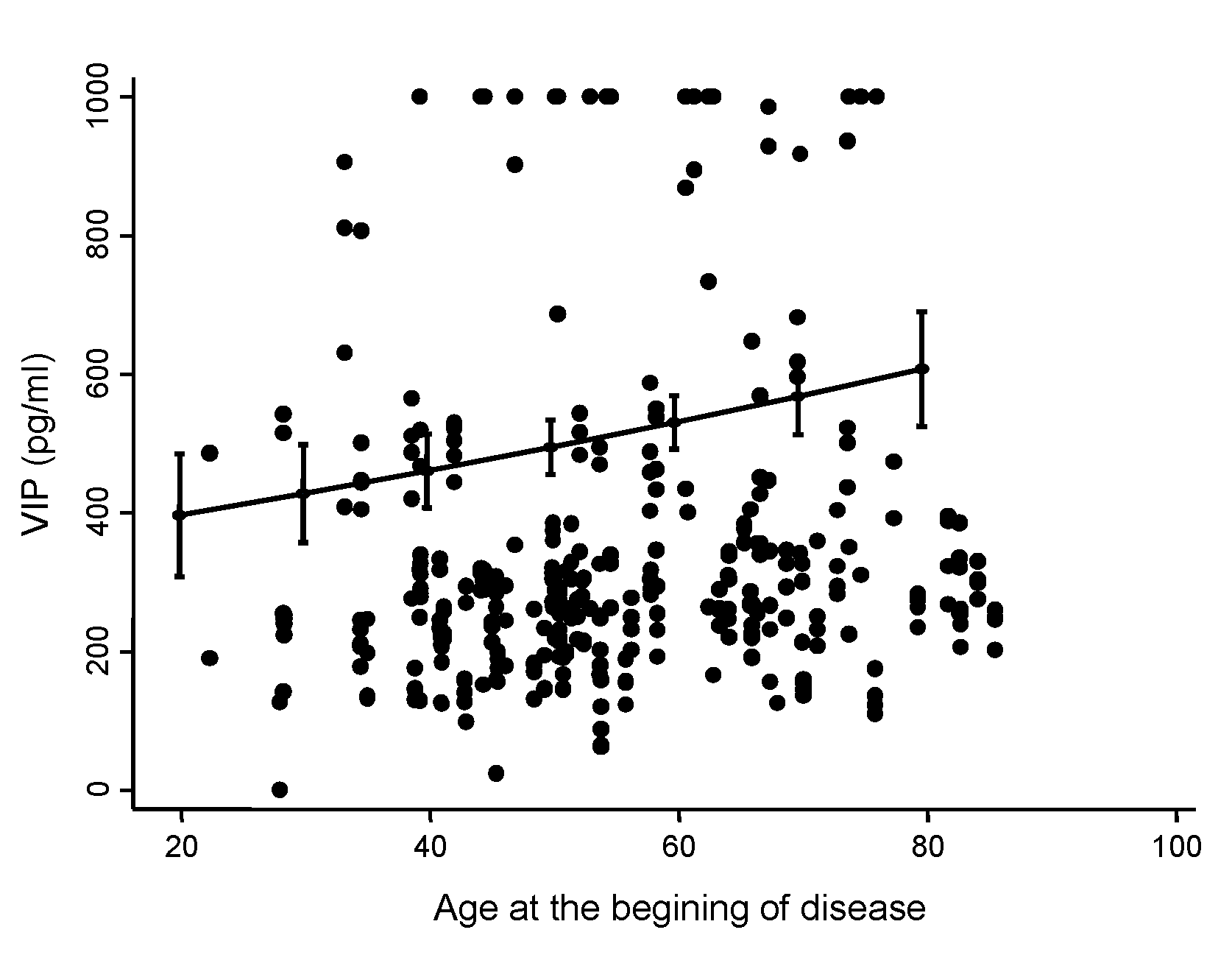

Supplement: Figure S3 — Correlation between age and VIP serum levels. Data are shown as dot plot and the exponential linear prediction with 95% confidence interval at ages 20, 30, 40, 50, 60, 70 and 80. These data were obtained using the command marginsplot of Stata 12 after performing the multivariable analysis displayed in Table 2 (dependent variable logarithmic transformation of censored VIP). (TIF) [file pone.0085248.s003.tif]
